# 

**Published:** 1843-05

**Authors:** 


### CONTENTS

OF NO. V.

### ORIGINAL COMMUNICATIONS.

### ESSAYS AND CASES.

Art. I.—Medical Topography of Central Arkansas; being Observations on the Locality, Climate, and Diseases of the City of Little Rock and vicinity in the year 1840. By W. J. Goulding, M.D., of Little Rock. - 321
Art. II.—Extraordinary Case of Pericarditis. By Chester G. Ballard, M. D., of Greencastle, Indiana. - 329
Art. III.—Miscellaneous cases and observations. Read before the Vincennes Medical Society, November, 1842. By Dr. J. S. Sawyer, of Vincennes, Indiana. - 332
Art. IV.—Cæsarean Operation on a Dwarf. By Cyrus Falconer, M. D., of Hamilton, Butler county, Ohio. 340
Art. V.—On the Use of Cathartics in Retention of the Placenta. By Dr. Thomas H. Todd, of Lincoln county, Tennessee. - - - - - - 345

### SELECTIONS FROM AMERICAN AND FOREIGN JOURNALS.

Presumption of Survivorship. - - - - - 352
Poisoning by Snails. - - - - - - 357
Electro-Magnetism in a Case of Poisoning, with Suggestions for its application to still-born Children, and some forms of Disease. - - - - - 357
Researches on Digestion. - - - - 365
The Treatment of Pneumonia in Guy's Hospital.—The pure Antimonial cure. - - - - 368
Communication of Pulmonary Air Vesicles by a direct route with the Pulmonary Veins. - - - 371
Luxation of the Patella on its axis. - - - 375
On Abnormal Nutrition (commonly called Inflammation), and on the mode in which its different Products are developed, as Softening, Suppuration, Granulation, Reorganization of Tissue, Morbid Growths, &c. &c. 376
Tubercular Deposits in the Bronchial Glands. - - 380
Pulmonary Consumption in Man and Animals. - - 381
Existence of Lymphatics in pseudo-memembranes. - 384
Local Treatment of Chancres by Sulphate of Copper and Cyanuret of Mercury. - - - - 385
Nitrate of Silver in bed Sores. - - - - 386
The Indian Hemp. - - - - - 386
Cure of Glanders in Man - - - - 388
Relative value of Quinine in large doses, as a remedy in Typhus. 386
Quinine in Intermittent and Remittent Fevers. - - 390
Remarkable case of Congenital Small-pox. - - 391
Dropsy of the Os Uteri. - - - - 392

ORIGINAL INTELLIGENCE.

Travelling Editorials. - - - - - - 393

